# Sources, Fate, and Detection of Dust-Associated Perfluoroalkyl and Polyfluoroalkyl Substances (PFAS): A Review

**DOI:** 10.3390/toxics11040335

**Published:** 2023-03-31

**Authors:** Usman M. Ismail, Haitham Elnakar, Muhammad Faizan Khan

**Affiliations:** 1 Department of Civil and Environmental Engineering, King Fahd University of Petroleum & Minerals, Dhahran 31261, Saudi Arabia; 2 Interdisciplinary Research Centre for Construction and Building Materials, King Fahd University of Petroleum and Minerals, Dhahran 31261, Saudi Arabia; 3 Alberta Environment and Protected Areas, Drinking Water and Wastewater, Regulatory Assurance Division, Government of Alberta, 2938 11 St. NE, Calgary, AB T2E 7L7, Canada

**Keywords:** PFAS, emerging contaminants, sand and dust storms, PFAS analytical techniques, dust-associated PFAS, PFAS toxicity

## Abstract

The occurrence of sand and dust storms (SDSs) is essential for the geochemical cycling of nutrients; however, it is considered a meteorological hazard common to arid regions because of the adverse impacts that SDSs brings with them. One common implication of SDSs is the transport and disposition of aerosols coated with anthropogenic contaminants. Studies have reported the presence of such contaminants in desert dust; however, similar findings related to ubiquitous emerging contaminants, such as per- and poly-fluoroalkyl substances (PFAS), have been relatively scarce in the literature. This article reviews and identifies the potential sources of dust-associated PFAS that can accumulate and spread across SDS-prone regions. Furthermore, PFAS exposure routes and their toxicity through bioaccumulation in rodents and mammals are discussed. The major challenge when dealing with emerging contaminants is their quantification and analysis from different environmental media, and these PFAS include known and unknown precursors that need to be quantified. Consequently, a review of various analytical methods capable of detecting different PFAS compounds embedded in various matrices is provided. This review will provide researchers with valuable information relevant to the presence, toxicity, and quantification of dust-associated PFAS to develop appropriate mitigation measures.

## 1. Introduction

Sand and dust storms (SDSs) are considered common meteorological hazards in areas with arid and semi-arid climates [1,2]. SDSs are accompanied by strong winds that lift large amounts of sand and dust, leading to visibility dropping to less than 1000 m. Blowing dust is less intense and causes visibility to reduce to a value between 1 and 10 km. When the visibility of a dust event is greater than 10 km, then the appropriate classification would be dust-in-suspension [1,3,4]. The adverse direct health implications of dust storms have been widely reported in different regions. A study by Chang et al. (2006) revealed that the number of visits to Shin Guang Memorial Hospital and National Taiwan University Hospital surged for three days after a severe dust storm, with children and cardiac patients as the major victims [5]. Inhalation of suspended dust can cause desert lung syndrome (silicosis) and trigger diseases such as bronchitis, asthma, chronic obstructive pulmonary disease, and emphysema, while dermal contact can result in conjunctivitis and dermatological problems [6]. The transportation and subsequent deposition of dust have major implications for climate change and the functioning of urban societies. The transboundary nature of SDSs makes it possible for dust from the Middle East and North Africa (MENA) region to reach glaciers in Afghanistan, and once deposited, it induces a warming effect by reducing the albedo of glaciers [7]. African dust has been found in the Western Caribbean and Florida, with major implications for air quality and aerosol atmospheric loading [8].

**Figure 1:**
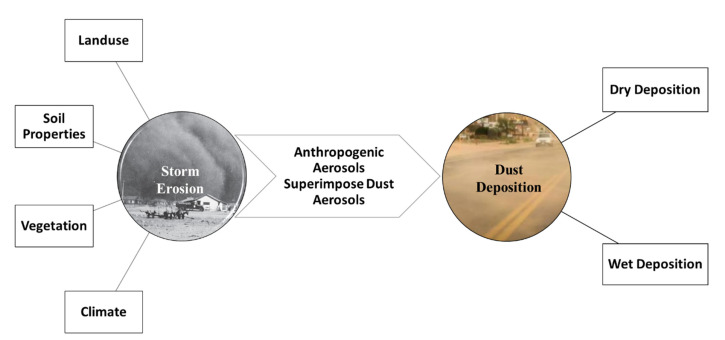
Key Factors Affecting Storm Erosion and the Principal Means of Dust Deposition.

One of the important, but scarcely studied, adverse environmental implications of SDS is aerosols coated by pollution originating from land-use changes and human-induced climate change. Figure 1 shows some of the key factors affecting storm erosion and the principal means of dust deposition. Anthropogenic aerosols can superimpose natural dust aerosols on dust storm transport routes, and they can be transferred several kilometers away [9]. It has been reported, for example, that arsenic and mercury have been detected in desert dust in downwind environments at concentrations higher than the regional crustal concentration [10]. The combined effect of these anthropogenic and dust aerosols may negatively influence downwind environments based on the frequency of exposure, the concentration and composition of particulates, and the health status of the receiving environment. Dust composition, accumulation, and potential consequences may vary due to weather conditions, traffic density, industrial activity, and proximity to mobile soil. There have been only rare studies about the characterization of falling dust in the Arabian Gulf region, mainly focused on reporting the pollutants within urban road dust, particularly trace metals [11,12,13,14,15,16,17,18,19]. While these studies represent a great start to the scientific effort to understand the anthropogenic pollutants mixed with street dust, there is still a need to advance our understanding to include emerging contaminants and study the impact of SDSs on baseline pollution levels.

## 2. Per- and Poly-Fluoroalkyl Substances (PFAS) Properties

Among the dust-associated emerging anthropogenic contaminants, per- and poly-fluoroalkyl substances (PFAS) are very persistent, with a half-life exceeding several decades [20]. The manufacturing and the use of PFAS, for a variety of purposes, started in the 1940s, and there is no consensus about the number of PFAS that have been produced, with 3000 PFAS reported as the smallest number [21]. Table 1 presents the chemical properties of some selected commonly used PFAS.

PFAS are very resistant to biological, chemical, and thermal degradation because of their strong carbon-fluorine bonds, and they can accumulate in the environment and ultimately bioaccumulate in living organisms [28,29]. It is important to note that several families and subfamilies of PFAS do exist, and the property of one PFAS may differ significantly from another in the length of the carbon chain, which is the major determinant of the chemical properties of PFAS. PFAS with at least seven carbon atom chains are considered long-chain PFAS, while those with fewer than seven carbon atom chains are considered short-chain PFAS [30]. The functional group of PFAS is also another important determinant of their properties [27]. While the use of long-chain and legacy PFAS, which are known to be more toxic and persistent, was slowly phased out in Europe and North America, the use of PFAS in less developed countries has not been prohibited. Furthermore, the production of PFAS precursors is on the rise as well [31,32].

## 3. Sources, Exposure Route, and Toxicity of Dust-Associated PFAS

### 3.1. Sources of Dust-Associated PFAS

PFAS are expected to accumulate in soil and potentially be picked up, transferred, and deposited during SDSs. To our knowledge, there are very limited studies available on the presence of PFAS in dust, runoff, and stormwater samples [33,34,35]. Particularly in Saudi Arabia and the Arabian Gulf Region, there is a lack of scientific evidence to support the extent to which PFAS are released and transported from the paved or unpaved ground surface, where they have accumulated, to stormwater or another location. Consequently, it is essential to understand the industries, facilities, and products that significantly release PFAS into the environment, so we can better identify the appropriate mitigation and adaptation measures against PFAS exposure [36].

#### 3.1.1. Firefighting Stations, Military Bases, and Aviation Sites

Aqueous film-forming foam (AFFF) is a PFAS-containing firefighting foam used to quickly extinguish fire, specifically class B fires, which are petroleum fuel based and can occur at military and aviation sites [37]. Depending on the formulation, AFFF may contain diverse types of PFAS [38]. The concentrations of 17 different PFAS in the dust matrix of 49 fire stations located in Canada and the United States have been compared with the concentrations of the same PFAS in the dust collected from 184 homes in the same region [38]. The most prevalent PFAS found in the dust matrices of both homes and firefighting stations were fluorotelomer alcohols (FTOHs) and di-poly-fluoroalkyl phosphoric acid esters (diPAPs), with a median concentration of at least 100 ng/g. It was also found that PFOS and PFOA concentrations were significantly higher in fire stations’ dust, even though 8:2 FTOH was significantly higher in dust obtained from homes. PFAS flame retardants probably originating from AFFF were also detected in oil sands process-affected water (OSPW), produced by the surface-mining activities as the oil sands industry in Alberta, Canada [39]. This finding emphasizes the increased risk of such toxic materials leaching into the groundwater if left untreated [40]. Another study conducted in the United States on different environmental media showed that the concentration of PFOS was the highest at 10 active US Air Force installations [41]. These studies confirmed that fire stations, aviation sites, and military site dust are significant sources of the widespread legacy PFAS.

#### 3.1.2. Fluorochemical Industry

The majority of global emissions of some specific PFAS is attributed to fluorochemical manufacturing sites, even though there are few of these sites around the world [42]. Releases from such facilities can impact a large population and have detrimental consequences for a vast geographical area. A study conducted by Hu et al. (2016) confirmed that there are only 16 fluorochemical manufacturing plants in the USA while Prevedouros et al. (2006) reported the existence of 33 fluoropolymer production plants worldwide, spread across North America, Europe, Japan, Russia, China, and India as of 2002 [43,44].

#### 3.1.3. Indoor Dust and Landfills

The term indoor dust is used here to provide an umbrella for a variety of PFAS-containing products used in daily household, office, and business activities. PFAS have been detected in jackets, carpets, personal care products, building materials, cleansers, polishes, office desks, food contact materials, upholstery, impregnation agents, and cars [45,46,47,48]. A detailed review by Savvaides et al. (2021) outlined the types of PFAS associated with many of the items listed above [49]. For example, FTOHs, PFCAs, PAPs, and PFSAs are often used in food packaging, as they have good resistance to water and oil. While this study seeks to find a connection between SDSs and PFAS-associated dust, it should be noted that some of these items are used daily and disposed of in open areas, which may expose them to SDSs. Other items have a significantly longer life; however, at the end of their life, they are disposed of in landfills, making the landfill another source of dust-associated PFAS. Chen et al. (2020) estimated that, in 2017, PFAS accumulated in landfilled carpets amounted to about 180 tons, while in-use carpets accumulated about 60 tons [50]. The concentration of PFAS in indoor dust may vary significantly from one location to another depending on the country’s wealth and development status. According to a study conducted by Shoeib et al. (2016), countries with high development indices, such as the USA have the highest median concentration of PFOS + PFOA in home dust, all exceeding 300 ng/g [31]. However, countries with stringent regulations regarding the use and consumption of PFAS, such as Norway, have a high human development index coupled with a very low median concentration (<50 ng/g) of PFOS + PFOA.

#### 3.1.4. Wastewater Treatment Plants

Wastewater treatment plants (WWTPs) often discharge treated effluents or bypass untreated or partially treated wastewater into rivers that may serve as source water for a variety of reuse purposes [51,52,53,54]. A study conducted by Shigei et al. (2020) investigated the presence and concentration of 20 targeted PFAS in water resources within the catchment area of the Zarqa river and also the buildup of PFAS in soils and crops [55]. The point of interest here is that PFAS can accumulate in the soil matrix, especially the topsoil, as there is potential for it to be carried by wind, resulting in dermal or inhalation PFAS exposure during dust events. The first finding revealed that WWTP effluent (14–24 ng/L) has a higher concentration than the influent (10–15 ng/L), indicating PFAS poor removal. This finding signifies that the WWTP may act as a point source for PFAS in the environment. PFAS were detected in the soil matrix albeit in a generally low concentration. A similar trend was reported by Dalahmeh et al. in Uganda [56]. Sludge from WWTPs is used as fertilizer, and a study conducted by Borthakur et al. (2022) confirmed the presence of PFCA in biosolids obtained from WWTPs in the USA, Canada, Australia, and Spain [20,57].

#### 3.1.5. Road Dust

Road dust is known to contain various types of contaminants that originate from vehicle exhaust emission, wear and tear of tires, litter, dust fall, accidental spills from vehicles transporting goods, biological debris, breakdown of particles from emission sources, and erosion as a result of water or wind from adjacent areas [58,59]. Several studies have confirmed that road dust contains contaminants such as polycyclic aromatic hydrocarbons (PAHs), pesticides, and metals [60,61]. In Saudi Arabia, numerous studies have confirmed the presence of heavy metals in road dust matrix, with a positive correlation with proximity to industrial sites that consume or generate trace metals [11,12,62,63]. However, the presence of PFAS in this dust matrix has not been investigated. A study conducted by Ahmadireskety et al. (2021) investigated the presence of 37 PFAS in street sweepings in the USA by collecting 117 sweeping samples and analyzing them [64]. More than 90% of the PFAS quantified were found to be perfluoroalkyl acids (PFAAs) and their precursors, and in one site, 26 different PFAS were found; other studies confirmed the presence of PFAS in roads [65,66].

### 3.2. PFAS’ Exposure Route

There are several routes by which humans and other animals may be exposed to PFAS, with the oral route being the most common. This exposure could happen through the intake of drinking water contaminated with PFAS, eating food associated with PFAS-containing products, and consumption of animal meat and plants in which PFAS have bioaccumulated. Exposure through inhalation of dust-associated PFAS, volatilized PFAS, and dermal absorption have been recorded, albeit to a lesser degree of frequency. Infants are exposed through breastmilk and in utero exposure from mothers exposed to PFAS [67,68,69,70,71]. The mechanisms of transfer between PFAS in the air and dust are yet to be understood; however, it is imperative to try and reduce dust-associated exposure, especially as it is more common in children and infants, who are more likely to inhale resuspended dust in great quantities [49]. It is known that atmospheric particulate matter functions as a sink that houses atmospheric contaminants, and associated contaminants are brought to the earth’s surface via dry deposition [59,72]. In the MENA region, dust storms transport a significant amount of dust, which could pose a significant risk for adults and older people, who are likely to be outside during dust events. While data about the exposure of individuals or animals to PFAS in the MENA region are generally not available, studies have been conducted in other regions. For instance, the US Centers for Disease Control and Prevention (CDC) reported the presence of PFAS in the blood samples of 98% of all Americans [73,74,75]. Another study by Geisy and Kannan (2001) sought to determine the global distribution of perfluorooctanesulfonate (PFOS) in wildlife by testing tissues and blood samples from mammals, fishes, reptiles, and birds in different countries [76]. Some selected findings from the study are reported as part of Table 2. Similarly, other studies have been conducted, albeit on a relatively smaller scale, and some of the significant findings are also reported in Table 2. The studies showed the ubiquitous nature of PFAS exposure, emphasizing the immediate need to advance hazard/exposure assessments for PFAS. Additionally, these findings highlight a gap in knowledge related to similar studies in ascertaining the exposure levels t associated with the population in developing countries, as in the case of the MENA region.

### 3.3. PFAS’ Toxicity

Research on potential human health risks due to PFAS exposure has mainly focused on the oral route, with limited data available on the health risks associated with dermal or inhalation exposure to PFAS [77]. Studies of health effects associated with PFAS exposure have mainly included long-chain perfluorooctanoic acid (PFOA) and PFOS because short-chain PFAS are thought to be less likely to bioaccumulate, more biodegradable, and less toxic, even though there are limited toxicity data available to back up the claims [77]. Sunderland et al. (2019) reviewed several studies related to the health implications of exposure to PFAS and found there was a significant correlation between elevated PFAS exposure and dyslipidemia, a metabolic disorder related to lipid profiles, such as total cholesterol and triglycerides [48]. In some studies, metabolic diseases, such as heart disease, overweight, diabetes, and obesity, were associated with PFAS exposure, although there are inconsistencies related to the evidence supporting such claims. The carcinogenicity of PFAS, immunotoxicity, and neurodevelopment deficiency have also been investigated, with most of these studies conducted on animals, such as rodents. Translating the results to human exposure tends to be very challenging because one of the main toxicity mechanisms of PFAS is peroxisome proliferation expression, which differs between humans and rodents. Nevertheless, studies conducted on rodents have shown that exposure to PFAS can cause liver disease, immune issues, thyroid disease, and cancer, as well as adverse effects on fetuses during pregnancy [78,79]. For these reasons, the US Environmental Protection Agency (US EPA) recently classified PFOA and PFOS as part of the fourth Contaminant Candidate List [77].

**Table 2:** Concentrations of some selected PFAS in organs and tissues of animals.

Class	Species	Location	Tissue	N	Year	PFOS	PFOSA	PFOA	PFHxS	Reference
Mammal	Human	Germany	Breast Milk	57	2006	0.028–0.309 [0.119]	–	–	–	[80]
		Gyor, Hungary		13	1996/1997	0.096–0.639 [0.330]	–	–	–	
		USA	Serum	1432	1999–2000	27.1–33.9 (30.3)	–	4.72–5.78 (5.22)	1.92–2.41 (2.15)	[81]
				1432	1999–2000	27.1–33.9 (30.3)	–	4.72–5.78 (5.22)	1.42–1.98 (1.67)	
				2120	2005–2006	16.0–18.2 (17.1)	–	3.48–4.42 (3.92)	1.51–1.82 (1.66)	
				2233	2009–2010	8.13–10.7 (9.32)	–	2.81–3.36 (3.07)		
				1954	2013–2014	4.53–5.54 (5.01)	–	1.76–2.15 (1.94)	0.996–1.18 (1.08)	
				1929	2017–2018	3.90–4.62 (4.25)	–	1.33–1.52 (1.42)		
		California, USA	Serum	96	2016	6.51	<LOD	1.41	0.767	[82]
				99	2017	7.47	<LOD	1.69	1.29	
	Melon-Headed Whale	Miyazaki, Japanese coast	Liver	12	1982	0.0053–0.0089 (0.0071)	6.0–12.3 (8.8)	–	–	[76,83,84]
		Ibaraki, Japanese coast		21	2001/2002	17.8–117 (51.1)	25.3–111 (65.8)	–	–	
	Indo-Pacific Humpback Dolphin	South China		10	2003–2007	26–693 (251)	9.51–37.6 (18.7)	0.243–8.32 (1.74)	–	
	Finless Porpoises			10	2003–2008	51.3–262 (151)	0.307–7.82 (1.70)	0.23–0.859 (0.33)	–	
	California Sea Lion	Coastal California		6	2001	<35–49	–	–	–	
	Polar Bear	Alaska, USA		17		180–680 (350)	–	–	–	
	Bottlenose Dolphin	Mediterranean Sea		5		170–430 (270)	–	–	–	
	Mink	Midwestern USA		18		970–3680 (2630)	–	–	–	
	Ringed Seal	Norwegian Arctic	Plasma	18		5–14 (9)	–	–	–	
Bird	Sea Gull	Rishiri Island, Hokkaido	Liver	14	1998	23–89 (53)	–	<19	–	[76,85]
	Common Cormorant	Sagami River, Kanagawa Prefecture		8	1999	170–650 (385)	–	<19	–	
	Black Tailed Gull	Korea	Liver	15	2001	70–500 (170)	–	–	–	
	Double-Crested Cormorant	Lake Winnipeg, Canada	Egg yolk	4		130–320 (210)	–	–	–	
	Common Cormorant	Italy	Liver	12		33–470 (96)	–	–	–	
	Bald Eagle	Midwestern USA	Plasma	26		1–2570 (360)	–	–	–	
Fish	Brown Trout	Michigan waters, USA	Eggs	3	2001	49–75 (64)	–	–	–	[76]
	Blue-Fin Tuna	Mediterranean Sea	Liver	8		21–87 (48)	–	–	–	
	Carp	Saginaw Bay, Michigan, USA	Muscle	10		60–300 (120)	–	–	–	
Reptile	Yellow-Blotched Map Turtle	Mississippi, USA	Liver	6	2001	39–700 (190)	–	–	–	
Amphibian	Green Frogs	Lake St. Clair, Michigan, USA	Plasma	5	2001	1–170 (72)	–	–	–	[76]

Key: (mean concentrations), [median concentrations], PFOS = Perfluorooctanesulfonic acid, PFOA = Perfluorooctanoic acid, PFOSA = Perfluorooctanesulfonamide. Note: For solid samples, wet weight is reported in ng/g, while liquid samples are reported in ng/mL.

## 4. Analytical Methods for the Detection and Quantification of PFAS

Owing to the omnipresent nature of PFAS and the wide range of concentrations reported in the literature, the detection of PFAS has been an arduous task for researchers. Sample preparation, detection methods, analytical instruments, and detection limits are not always provided in detail and vary greatly among researchers and commercial laboratories around the world. Even though dust is our major concern, a detailed list of some of the analytical methods published in peer-reviewed journals for the detection of PFAS in various matrices has been compiled and is provided in Table 3. The purpose of Table 3 is to provide researchers with an overview of which instruments are capable of quantifying PFAS and their precursors from specific matrices and their detection limits. As shown in Table 3, PFAS have been identified extensively in different regions, including North America, Europe, China, and Australia. In these studies, PFAS were extracted from drinking water, wastewater, atmosphere, dust particulates, street sweeping, surface water, synthetic contaminated water/soils, sediments, contaminated soil, and groundwater from various locations, AFFF-impacted soil and groundwater, landfill and leachate, among other samples. In general, the pretreatment steps are based on the sample matrices but typically include solid phase extraction (SPE) with Oasis WAX, HLB, or Strata-X cartridges.

PFAS in water and soil samples are usually detected using liquid chromatography coupled with mass spectroscopy (LC-MS) or tandem mass spectrometry (LC-MS/MS). Similarly, high-performance or ultra-high-performance liquid chromatography (HPLC or UHPLC) was also employed by many researchers, as shown in Table 3. In terms of mass spectrometry, the most commonly reported spectrometers for PFAS also included triple quadrupole (QqQ), quadrupole time-of-flight (QTOF), and quadrupole-orbitrap in positive or negative electrospray ionization (ESI) modes [86,87,88,89]. Typically for atmospheric or dust samples, gas chromatography mass spectrometry (GCMS) equipped with electron impact (EI) [38], electron capture negative ion (ECNI) [90], and mass selector detector (MSD) [91] have been used by researchers. Despite these studies, researchers are constantly investigating new detection methods, as there might be still many unknown PFAS precursors in the environment. Usually, targeted analysis is performed to quantify known PFAS and their precursors. The targeted analysis includes methods that are only applicable to detect and measure known analytes in the matrix. The analytical standard for quantification already exists in targeted analysis. In contrast, non-targeted analysis includes methods that detect known and unknown analytes. Typically, high-resolution mass spectrometry (HRMS) is used for non-targeted analysis, as employed by [92] to predict the fate of new generations of PFAS. These instruments are capable of measuring both known and unknown analytes, and data can be stored in HRMS for analyzing new analytes later. Some of the new detection methods recently developed used particle-induced gamma ray emission spectroscopy (PIGE) [93,94] and fluorine nuclear magnetic resonance (19F-NMR) [95]. The limit of detection (LOD), the limit of quantification (LOQ), the method detection limit (MDL), and the method quantification limit (MQL) varied among samples from water, soil, and air/dust, as shown in Table 3.

**Table 3:** Analytical methods used in the detection of PFAS (precursors of PFCAs, PFSAs, sulfonamide, and telomers) in various matrices.

Matrix	Location	Target Application	Detection Equipment	Detection Limit	Reference
**Dust**					
House and fire station dust	USA and Canada	Presence of PFAS in a fire station and house dust	HPLC-ESI-MS/MS and GC/EI-MS	MDL in ng/g dust for house and fire station dust: 6:2 diPAP = 0.48, 2.54; 8:2 diPAP = 10.63, 9.63; PFCAs = 0.06–15.80, 0.47–48.90; PFSAs = 0.20–22.28, 0.97–8.56; PFPA = 0.14, 1.2.	[38]
Fire station dust	Massachusetts, USA	Presence of PFAS in a fire station and house dust	PIGE spectroscopy for total fluorine; for targeted analysis, LC-MS/MS	MDL for total fluorine was 25 µg/g. PFAS MDLS ranged from 0.00242 to 18.1 ng/g	[94]
College dust	USA	PFAS in college dust	GC–ECNI/MS and GC–EI/MS or by LC–MS/MS	LOQ in ng/g: PFCAs = 20, PFSAs = 20	[90]
House dust	Belgium, Italy and Spain	PFAS in house dust and human exposure to PFAS	HPLC-MS/MS	LOQ in ng/g: PFCAs = 0.02–0.27; PFSAs = 0.003–0.57.	[96]
Indoor dust from urban, industrial, and e-waste dismantling areas	Guangdong Province, China	Detection of PFAS in indoor dust of different indoor facilities	HPLC-MS/MS	LOQ of analytes ranged from 0.02–0.50 ng/mL (liquid extracts were used for detection limit quantification).	[97]
Street sweepings	Across the USA	Detection of PFAS in street sweepings	UHPLC-MS/MS	MDL, MQL in ng/g dust: PFCAs = 0.01–0.02, 0.01–0.69; PFSAs = 0.01–0.13, 0.03–0.42; PFPAs = 0.03–0.12, 0.10–0.41; HFPO-DA = 0.41, 1.37; FHEA = 0.08, 0.26; FOEA = 0.04, 0.13; FOUEA = 0.01, 0.05; FDUEA = 0.01, 0.03; N-6:6 PFPi = 0.01, 0.04; diSAmPAP = 0.04, 0.12; EtFHxSE (SYN 2) = 0.40, 1.33.	[64]
Dust from university buildings	USA	To confirm the implementation of “healthier material” manufactured without PFAS in buildings	HPLC-ESI-MS/MS	MQL, MDL in ng/g: PFCAs = 0.05–9.19, 0.02–2.76; PFSAs = 0.05–15.06, 0.02–4.52; NaDONA = 0.10, 0.03.	[98]
**Soil and Groundwater**					
Artificially contaminated soil	N/A	Soil remediation	LC-TQMS	IDL = 0.0001 mg/L(Soil slurry was analyzed)	[99]
Soil sample	Germany	The optimized fast and simple extraction method for PFAS	HR–CS–GFMAS	-	[100]
AFFF-impacted soil	USA	New methods for PFAS detection in soil	LC-QTOF-MS	-	[89]
Soil samples near industrial areas	Shifang City, China	Determination of PFAS in soil	LC-MS/MS Qtrap with negative ion ESI	LODs, LOQs in ng/g: PFCAs = 0.001–0.006, 0.004–0.018; PFSAs = 0.001–0.01, 0.004–0.034	[101]
Surface soil	New Jersey, USA	Predicting the fate of new generation PFAS	HRUPLC-QtoF MS in –ve ESI for non-targeted analysis; LC-MS/MS for targeted analysis.	-	[92]
Soil and groundwater samples	Military bases in Pennsylvania and Michigan impacted by AFFF	Soil and groundwater remediation-electron beam technology	LC-MS/MS	Detection limits ranged from 0.3 ng/g dry weight to 1.2 ng/g dry weight.	[102]
Groundwater impacted by AFFF use	Willow Grove, Pennsylvania, USA	Treatment of contaminated groundwater	LC-MS/MS QTOF or QTRAP	LOD between 0.1 To 10 ng/L, MDL between 5000 to 10,000 ng/L	[103]
Landfill leachate	Queensland, Australia	Foam-fractionation (water treatment)	HPLC-MS/MS	PFCAs and PFSAs LOD = 0.02–0.05 µg/L, PFCAs and PFSAs LOQ = 0.08–0.17 µg/L, 6:2 FTS LOD = 0.03 µg/L, 6:2 FTS LOQ = 0.1 µg/L; PFECHS LOD = 0.03 µg/L, PFECHS LOQ 0.11 µg/L.	[86]
Synthetic soils including clay and sand	N/A	Treatment of contaminated soil pyrolysis and thermal air degradation	UPLC. Thermal desorption−pyrolysis (TD−Pyr) connected to a GC−MS to detect products of PFOA and PFOS	The limits of detection (S/N = 3) are 7 nmol/L for PFBA and PFPeA, 2 nmol/L for PFSAs, 5 nmol/L for other anionic PFAS, and 5 nmol/L for cationic and zwitterionic PFAS (Liquid extracts were used for detection limit quantification).	[104]
Soil samples near industrial areas, airports, landfills, and fire stations	Shanghai, China	Detection of PFAS contamination and distribution	LC-MS equipped with C18 column	MDL, MQL in µg/kg (dry weight): PFPrA = 0.05, 0.15; PFCAs = 0.02–0.1, 0.01–0.3; PFSAs = 0.003–0.03, 0.01–0.10; HFPO-DA = 0.003, 0.01.	[105]
Biosolid extract and clean extract	USA	New PFAS detection method	19F-NMR spectroscopy	A detection limit of 50 nM (25 µg/L for PFOS) was achieved in groundwater samples	[95]
AFFF impacted groundwater	Colorado, US	Water treatment-NF and UV-sulfite treatment train	LC-QtoF-MS	LOQ of groundwater in ng/L: PFCAs = 0.7; PFSAs = 0.4–0.7; FhxSA = 0.4, 6:2 FTS = 0.7.	[106]
**Water**					
Water	N/A	Water treatment-electro oxidation	LC-MS/MS	LOD 1.0 ng/L, LOQ 3.0–4.0 ng/L	[107]
River water	Pretoria, South Africa	A new extraction method for PFAS in water	HPLC-DAD and UHPLC-MS/MS	LOD and LOQ for HPLC in ng/L = 0.3–0.66, 1.0–2.2; LOD and LOQ for UHPLC in ng/L = 0.011–0.04, 0.037–0.12	[108]
Model and industrial wastewater	N/A	Water treatment-electrochemical oxidation	LC-MS/MS with negative ESI	MRL is 2 ng/L for all samples	[109]
Simulated water	N/A	Water treatment-UV photo-catalysis	HPLC-MS equipped with MicroToF MS	PFOA = 1 mg/L, fluoride = 0.1 mg/L, HPLC ToF = 0.01 mg/L	[110]
Ultrapure water and river water	Grand River water, Canada	Drinking water treatment-ion-exchange	GC/MS	MDL, LOQ for ultrapure water in ng/L = 11–23, 35–74; MDL, LOQ for river water in ng/L = 16–49, 52–157.	[111]
Surface water	Alabama, USA	Spatial distribution of PFAS in surface water	UHPLC-MS/MS equipped with ESI	LOD in ng/L: PFCAs = 0.21–0.72; PFSAs = 0.69–1.27; HFPO-DA = 0.55; ADONA = 0.48; PF4OpeA = 0.91; PF5OhxA = 0.75; 3,6-OPFHpA = 0.69	[112]
Drinking water	China	Drinking water treatment, adsorption	HPLC-TQ MS	PFCAs LOD = 0.01–0.1 ng/L, PFCAs LOQ = 0.05–0.1 ng/L, PFSAs LOD = 0.02–0.05 ng/L, PFSAs LOQ = 0.05–0.12 ng/L	[87]
Drinking water, surface water, and wastewater	Thessaloniki WTP, Greece	PFAS analysis technique and workflow	Orbitrap Q ExactiveTM Focus equipped with HESI-II	MQL in ng/L. IDL and IQL in µg/L PFSAs = 0.0011–0.2063, 0.02–0.17, 0.02–0.56; PFCAs = 0.0024–0.2605, 0.02–0.22, 0.05–0.71.	[113]
Drinking water samples	Multiple locations in USA	New extraction method for PFAS in water	HPLC-MS/MS	LOD in ng/L: PFCAs = 0.08–0.30; PFSAs = 0.05–0.30.	[114]
Surface water	Netherlands	Determination of PFAS in surface waters and validation of new analytical methods	UHPLC-MS/MS connected to a Sciex Qtrap 5500	I-LOD = 0.01–0.09 ng/mL; I-LOQ = 0.03–0.30 ng/mL; MDL = 0.02–0.75 ng/L; MQL = 0.07–2.55 ng/L.	[115]
Municipal wastewater samples	Northern New Jersey, USA	Rapid analytical method for PFAS quantification	HPLC-TQMS and Nano-ESI-HRMS	LODs in ng/L first for Nano-ESI HRMS and then for HPLC-TQMS: PFSAs = 4.2–25.1, 1.1–135.6; PFCAs = 3.2–36.2, 6.7–87.8; GenX = 6.4, 96.6	[116]
Surface water	Poyang Lake, China	Distribution, partitioning behavior, and flux of PFAS	UPLC TQ in negative ESI	MQL, MDL in pg/L: PFCAs = 0.41–18, 0.81–39; PFSAs = 1.4–33, 2.0–35; 6:2 Cl-PFESA = 1.8, 15, 8:2 Cl-PFESA = 1.0, 3.6; OBS = 2.0, 92; PF4OpeA = 6.8, 3.1; PF5OhxA = 3.0, 2.7; NaDONA = 3.0, 4.6; PFEESA = 0.94, n.d; HFPO-DA 6.9, 7.0, HFPO-TA = 11, 71	[117]
AFFF-impacted stormwater	USA	Stormwater treatment-photocatalysis	LC-QtoF-MS. ICS-90 for fluoride; UHPLC for the analyte	MDL in ng/L for column: PFCAs = 400–950; PFSAs = 100–250.	[118]
Surface and tap waters	Biscayne Bay, USA	Presence of PFAS in water	LC-MS/MS system equipped with AJS ESI source	MDL, IQL in ng/L: PFCAs = 0.01–1.99, 0.26–205; PFSAs = 0.01–0.45, 0.51–97.3; Adona = 0.02, 0.98; GenX = 0.02, 5.90; PFOUDS = 0.36, 66.00.	[119]
Wastewater treatment plant influent	South-East Queensland, Australia	The trend of PFAS in WWTP influent	HPLC-MS/MS using TurboIonSpray^®^ probe	PFCAs = 0.27–8.7; PFSAs = 0.54–32.0; ADONA = 1.0, PFECHS = 0.47, 6:2FTAB = 5.4, 8-Cl-PFOS = 1.0, PFOPA = 1.8.	[120]
Drinking water source	Tianjin City, China	Detection of PFAS in lakes.	HPLC-MS/MS in negative ESI mode	LOD, LOQ in ng/L: PFCAs = 0.02–0.14, 0.05–0.47; PFSAs = 0.00–0.03, 0.01–0.11; 8:2Cl-PFESA = 0.00, 0.01; 6:2Cl-PFESA = 0.00, 0.01; PFECHS = 0.00, 0.01; HFPO-DA = 0.08, 0.28.	[121]
Spiked synthetic tap water	University of Notre Dame, USA	A new method for PFAS screening	PIGE spectroscopy	LOD in ppt F, LOD in ppt analyte, HFPO-DA = 48.8, 77.1; PFSAs = 31.5–64.7, 34.6–59.4; PFCAs = 31.6–40, 45.9–58.8.	[93]
**Air**					
Air samples and carpets	Southern Rhode Island, USA	To study the partitioning of volatile PFAS between air, dust, and carpet	GC-MSD operating in positive chemical ionization mode using selected ion monitoring	LOQ in (ng/uL): PFCAs = 0.01–0.07; PFSAs = 0.002–0.20; 8:2 FTAcr = 0.01, 10:2 FTAcr = 0.02.	[91]
Air matrix	42 developing countries	Monitoring of air quality	LC–MS/MS with negative ESI mode	LOQ was the lowest point in the calibration curve since target compounds were not detected.	[122]
Fine airborne particulate matter (PM2.5)	Dublin and Enniscorthy, Ireland	Screening of atmospheric PM	On-line SPE LC-HRMS	LOD, LOQ in pg/mL: PFCAs = 0.17–0.51, 0.58–1.69; PFSAs = 0.08–0.17, 0.26–0.58.	[123]
Atmosphere	Japan	Detection of PFAS in the atmosphere	Combustion ion chromatography (CIC), HPIC, ILC-TQ MS with negative ESI and GCMS	IDL in pg, LOQ in pg, LOQ in pg/m^3^: PFSAs = 0.003–3.67, 1.77–500, 0.033–9.43; PFCAs = 0.001–5.88, 1.89–500, 0.036–18.9; TFA = 0.836,200, 3.77; FTIs = 0.039–4.72, 50–1000, 0.943–18.9; PFDeI = 1.20, 250, 4.72, PFDoI = 1.72, 250, 4.72 PFBuDil = 0.103, 50.0, 0.943, PFHxDil = 0.246, 50.0, 0.943, PFODil = 1.07, 250, 4.7; 6:2 Cl-PFESA = 0.005, 1.86, 0.035, HFPO-DA = 0.007, 2.00, 0.038	[124]

KEY: MDL = method detection limit, MQL = method quantification limit, LOD = limit of detection, LOQ = limit of quantification, IDL = instrument detection, IQL = instrument quantification limit, MRL = method reporting limit, I-LOD = instrument limit of detection, I-LOQ = instrument limit of quantification. Abbreviations of the different precursors detected can be found in the Supplementary Materials.

## 5. Conclusions

SDSs are naturally occurring phenomena peculiar to arid and semi-arid regions. They are transboundary in nature, and as such, they are important for the global biogeochemical cycling of nutrients, which is essential for agriculture and the fertilization of oceans. However, with climate change leading to extensive droughts and loss of vegetation in many regions, the frequency at which they occur may significantly change which will result in negative consequences significantly outweighing their positive implications. SDSs may pick up contaminants along their journey, which will lead to human exposure through inhalation or dermal contact. Some contaminants, such as heavy metals and polyaromatic hydrocarbons (PAHs), have been well studied, and their presence in the dust of different regions has been confirmed. However, there are emerging contaminants such as PFAS, the presence of which in the dust matrix has only been scarcely studied. PFAS are a group of more than 3000 persistent fluorinated organic compounds with a half-life exceeding several decades. Because of their specific characteristics, they are used in various applications; hence, they have numerous dust-associated sources in the environment, including firefighting stations, military bases, indoor dust, the atmosphere, landfills, and water and wastewater treatment plants. The health risks associated with PFAS exposure are yet to be fully understood; however, several studies conducted on rodents have indicated carcinogenicity, immunotoxicity, and the potential for PFAS to cause neurodevelopmental disorders. The above uncertainties make the presence of PFAS in the dust matrix worth exploring and make it more of a concern, especially in arid and semi-arid regions. However, it must be noted that the major challenge when dealing with PFAS contamination is the lack of consensus about standard analytical techniques and sample preparation procedures. This review provided a summary of analytical instruments and their detection limits used to quantify different PFAS and their precursors from various environmental media. More studies are recommended to develop new PFAS detection methods to curb PFAS exposure effectively, especially in matrices considered complex, such as SDS.

## Figures and Tables

**Table 1:** Chemical properties of some selected PFAS.

PFAS Name		Acronym	Molecular Formula, Wt (g/mol)	Solubility (mg/L) (25 °C)	Melting Point (°C)	Boiling Point (°C)	Density (g/cm^3^)	Vapor Pressure (Pa)	References
**Perfluoroalkyl Substances**	**Perfluorocarboxylic Acids (PFCAs)**								
	Perfluorobutanoic acid	PFBA	C_3_F_7_COOH, 214.0	49,000	−17.5	121	1.651 (20 °C)	251 (25 °C)	[22,23]
	Perfluoropentanoic acid	PFPeA	C_4_F_9_COOH, 264.1	9812	−13.2–−5.9	–	1.713 (25 °C)	151 (25 °C)	[23,24]
	Perfluorohexanoic acid	PFHxA	C_5_F_11_COOH, 314.1	1893	7.8–14.8	–	1.759 (20 °C)	13.2 (25 °C)	
	Perfluoroheptanoic acid	PFHpA	C_6_F_13_COOH, 364.1	356	19.1–32.8	–	–	55 (25 °C)	
	Perfluorooctanoic acid	PFOA	C_7_F_15_COOH, 414.1	4340 (24.1 °C)	44.8–52.3	–	1.8	2.0 (25 °C)	[24,25]
	**Perfluorosulfonic Acids (PFSAs)**								
	Perfluorobutane sulfonic acid	PFBS	C_4_F_9_SO_3_H, 300.1	6875	20.4–70.4	80–211	1.81–1.85	132 (25 °C)	[23,26]
	Perfluorohexane sulfonic acid	PFHxS	C_6_F_13_SO_3_H, 400.1	236	190	95–452	1.84	47.9 (25 °C)	[23,24]
	Perfluorooctane sulfonic acid	PFOS	C_8_F_17_SO_3_H, 500.1	7.7	15.2–185	133–249	1.84–1.85	16.98 (25 °C)	
**Polyfluoroalkyl Substances**	**Fluorotelomer Carboxylic Acids (FTCAs)**								
	6:2 Fluorotelomer carboxylic acid	6:2 FTCA	C_6_F_13_CH_2_COOH, 378.1	559	55	175–193	1.64–1.67	5.8	[26,27]
	**Fluorotelomer Sulfonic Acids (FTSAs)**								
	4:2 Fluorotelomer sulfonic acid	4:2 FTSA	C_4_F_9_(CH_2_)_2_SO_3_H, 328.2	28000	107	216	1.68	0.33	[26,27]
	6:2 Fluorotelomer sulfonic acid	6:2 FTSA	C_6_F_13_(CH_2_)_2_SO_3_H, 528.2	1323	18.7–80.7	219–272	1.64–1.71	0.01	
	8:2 Fluorotelomer sulfonic acid	8:2 FTSA	C_8_F_17_(CH_2_)_2_SO_3_H, 528.2	58	16.8–91.1	224–293	1.69	0.01	
	**Fluorotelomer Alcohols (FTOHs)**								
	4:2 Fluorotelomer alcohol	4:2 FTOH	C_4_F_9_(CH_2_)_2_OH, 264.1	2703	–	137.5	–	105 (25 °C)	[23,25]
	6:2 Fluorotelomer alcohol	6:2 FTOH	C_6_F_13_(CH_2_)_2_OH, 364.1	98	–	171.5–173.5	–	38 (25 °C)	[26,27]
	8:2 Fluorotelomer alcohol	8:2 FTOH	C_8_F_17_(CH_2_)_2_OH, 464.1	3.2	44.75–46.95	201.3–202.0	–	13.5 (25 °C)	[24,26,27]
	10:2 Fluorotelomer alcohol	10:2 FTOH	C_10_F_21_(CH_2_)_2_OH, 564.1	0.10	89.75–92.35	228.4	–	4.90 (25 °C)	

## Data Availability

No new data were collected or analyzed in this study. Data sharing is not applicable to this article.

## Supplementary Materials

The following supporting information can be downloaded at: https://www.mdpi.com/article/10.3390/toxics11040335/s1. Table S1: Literature review of the analytical methods used in the detection of PFAS in various matrices, including the name of the specific PFAS tested.
