# Supplementary material for: Sources, Fate, and Detection of Dust-Associated Perfluoroalkyl and Polyfluoroalkyl Substances (PFAS): A Review
Source: Toxics. 2023 Mar 31;11(4):335. doi: 10.3390/toxics11040335 (PMC10146191; doi:10.3390/toxics11040335)
Supplement: Supplementary file 1 [file toxics-11-00335-s001.zip › toxics-2294321-supplementary.pdf]

Table S1. Literature review of the analytical methods used in the detection of PFASs in various matrices including the name of the specific PFAS tested.

**Chemical Compound/Derivative**	**Matrix**	**Location**	**Target Application**	**Detection Equipment**	**Detection Limit**	**Reference**
PFOA and PFOS	Artificially contaminated soil		Soil remediation	LC- TQMS	IDL = 0.001 mg/L	(Kewalramani et al., 2022)
PFOA and PFOS	Water		Water treatment-electro oxidation	LC-MS/MS	LOD 1.0 ng/L, LOQ 3.0-4.0 ng/L	(Hwang et al., 2022)
PFOA and PFOS	Synthetic groundwater		Groundwater treatment-membranes	LCMS	Linear dynamic range between 0.5 to 50 ug/L was used for experiment	(Wan, Mills, Wang, et al., 2022)
PFOA	Synthetic water (Ultrapure water)		Water treatment-Hydrated electron degradation on ion-exchange resin	HPLC-MS/MS	The detection limit of PFOA was 0.1 μg/L.	(Cui & Deng, 2022)
PFOA, PFDA, PFTeDA, PFOS	River water	Pretoria, South Africa	New extraction method for PFASs in water	HPLC-DAD and UHPLC-MS/MS	LOD, LOQ for HPLC and UHPLC in ng/L<br>PFOA = 0.3, 1.0 and 0.04, 0.12; PFDA = 0.41, 1.4 and 0.02, 0.07; PFTeDA = 0.46,1.5 and 0.011, 0.037; PFOS = 0.66, 2.2 and 0.03,0.10	(Selahle et al., 2022)
PFBA, PFPeA, HFPO-DA, PFOA, PFOS	Synthetic water and lake water		Water treatment-adsorption	LC-MS		(Wan, Mills, Qu, et al., 2022)

C4-PFBA, C6-PFHxA, C8-PFOA, C6-PFHxS, C8-PFOS, C4-PFBS	Model and industrial wastewaters		Water treatment- electrochemical oxidation	LC-MS/MS with -ive ESI	MRL is 2 ng/L for all samples	(Nienhauser et al., 2022)
Na-TFMS, K-PFBS, K-PFHxS, PFOS, Na-TFA, PFOA, HFPO-DA, PFPD, PFOPA, 8:2-FTOH, PTFE, PFA, 4-FBA, TOLYLFL	Soil and sewage sludge	Germany	New PFASs detection method	Fluorine K-edge XANES spectra on the PHOENIX II beamline. CIC used for TF/EOF analysis.	F-XANES can be used to identify PFOS down to 10 mg/L. LOD and LOQ for fluoride are 1 and 2 ug/L, respectively.	(Roesch et al., 2022)
PFOA and short chain by-products	Simulated water	-	Water treatment- UV photo-catalysis	HPLC-MS equipped with MicroToF MS	PFOA = 1 mg/L, fluoride = 0.1 mg/L, HPLC ToF = 0.01 mg/L	(Duan et al., 2022)
PFHpA, PFOA and PFNA	Ultrapure water and river water	Grand River water, Canada	Drinking water treatment- ion-exchange	GC/MS	MDL, LOQ for ultrapure water in ng/L (PFHpA = 23, 74, PFOA = 11, 35, PFNA = 16, 51)<br>MDL, LOQ for river water in ng/L (PFHpA = 16, 52, PFOA = 20, 65, PFNA = 49, 157).	(Rahman et al., 2022)
PFBS, PFPeA, PFOA	Soil sample	Germany	Optimized fast and simple extraction method for PFAS	HR–CS–GFMAS	-	(Simon et al., 2022)
M8PFOA, M2PFOA, M3PFHxS, M8PFOS, d-NMeFOSA, d-NEtFOSA, d9-	Air matrix	42 developing countries	Monitoring of air quality	LC–MS/MS with –ve ESI mode	LOQ was the lowest point in the calibration curve since target compounds were not detected.	(Camoiras González et al., 2021)

NEtFOSA, d7-NMeFOSE, M8FOSA, MPFHxS, MPFOS.						
PFCAs, PFSAs, HFPO-DA, ADONA, PF4OpeA, PF5OhxA, 3,6-OPFHpA, MPFOS	Surface water	Alabama. USA	Spatial distribution of PFASs in surface water	UHPLC-MS/MS equipped with ESI	LOD in ng/L: PFCAs = 0.21 – 0.72; PFSAs = 0.69 – 1.27; HFPO-DA = 0.55; ADONA = 0.48; PF4OpeA = 0.91; PF5OhxA = 0.75; 3,6-OPFHpA = 0.69	(Viticoski et al., 2022)
PFBS, PFHxS[1], PFOS[1], PFCAs precursors, HFPO-DA, ADONA, 9C1-PF3ONS, NMeFOSAA[1], NEtFOSAA[1], 11C1-PF3OudS,	DWTP influent and effluent	New York, USA	Drinking water treatment- advanced oxidation process	LC-MS/MS equipped with ESI	MDL in ng/L: PFCAs = 0.04 – 0.39; PFBS = 0.32; PFHxS[1] =0.14; PFOS[1] = 0.08; HFPO-DA =0.12; ADONA = 0.04; 9C1-PF3ONS = 0.09; NMeFOSAA* = 0.10; NEtFOSAA* = 0.13; 11C1-PF3OudS = 0.07	(Venkatesan et al., 2022)
PFCAs and PFSAs precursors	Drinking water	-	Drinking water treatment, adsorption	HPLC- TQ MS	PFCAs LOD = 0.01 – 0.1 ng/L, PFCAs LOQ = 0.05 – 0.1 ng/L, PFSAs LOD = 0.02 – 0.05 ng/L, PFSAs LOQ = 0.05 – 0.12 ng/L	(R. Chen et al., 2022)
PFCAs and PFSAs precursors	AFFF-impacted soil	USA	New methods for PFASs detection in soil	LC-QTOF-MS	-	(Shojaei et al., 2022)
PFCAs and PFSAs precursors	Soil sample near industrial area	Shifang City, China	Determination of PFASs in soil	LC-MS/MS Qtrap with negative ion ESI	LODs, LOQs in ng/g: PFCAs = 0.001 – 0.006, 0.004 – 0.018; PFSAs = 0.001 – 0.01, 0.004 – 0.034	(Gan et al., 2022)

PFCAs and PFSAs precursors	fine airborne particulate matter (PM2.5)	Dublin and Enniscorthy, Ireland	Screening of atmospheric PM	On-line SPE LC-HRMS	LOD, LOQ in pg/mL: PFCAs = 0.17 – 0.51, 0.58 – 1.69; PFSAs = 0.08 – 0.17, 0.26 – 0.58.	(Kourtchev et al., 2022)
PFCAs and PFSAs precursors	Drinking water, surface water and wastewater	Thessaloniki WTP, Greece	PFASs analysis technique and workflow	Orbitrap Q ExactiveTM Focus equipped with HESI-II	MQL in ng/L. IDL and IQL in ug/L<br>PFSAs = 0.0011 – 0.2063, 0.02 – 0.17, 0.02 – 0.56; PFCAs = 0.0024 – 0.2605, 0.02 – 0.22, 0.05 – 0.71.	(Koronaiou et al., 2022)
PFCAs and PFSAs precursors	Drinking water samples	Multiple locations in US	New extraction method for PFASs in water	HPLC-MS/MS	LOD in ng/L: PFCAs = 0.08 – 0.30; PFSAs = 0.05 – 0.30.	(Skaggs & Logue, 2021)
PFCAs and PFSAs precursors	soil and groundwater samples	Military bases in Pennsylvania and Michigan impacted by AFFF[2]	soil and groundwater remediation- electron beam technology	LC-MS/MS	Detection limits ranged from 0.3 ng/g dry weight to 1.2 ng/g dry weight.	(Lassalle et al., 2021)
PFCAs and PFSAs precursors	Fire station dust	assachusetts, USA	Presence of PFASs in fire station and house dust	PIGE spectroscopy for total fluorine. For targeted analysis LC-MS/MS	MDL for total fluorine was 25 ug/g. PFASs MDLS ranged from 0.00242 to 18.1 ng/g	(Young et al., 2021)

PFCAs and PFSAs precursors	Colleges dust	USA	PFASs in college dust	GC–ECNI/MS and GC–EI/MS or by LC–MS/MS	LOQ in ng/g: PFCAs = 20, PFSAs = 20	(Schildroth et al., 2022)
PFCAs and PFSAs precursors	House dust	Belgium, Italy and Spain	PFASs in house dust and human exposure to PFAS	HPLC-MS/MS	LOQ in ng/g: PFCAs = 0.02 – 0.27; PFSAs = 0.003 – 0.57.	(de la Torre et al., 2019)
PFCA precursors	Surface water	Netherlands	Determination of PFASs in surface waters and validation of new analytical methods	UHPLC-MS/MS connected to a Sciex Qtrap 5500	I-LOD = 0.01 – 0.09 ng/ml; I-LOQ = 0.03 – 0.30 ng/ml; MDL = 0.02 – 0.75 ng/L; MQL = 0.07 – 2.55 ng/L.	(Awchi et al., 2022)
PFCAs, PFSAs	Groundwater impacted by AFFF use	Willow Grove, PA, USA	Treatment of contaminated groundwater	LC-MS/MS QTOF or QTRAP	LOD between 0.1 To 10 ng/L, MDL between 5000 to 10000 ng/L	(Ellis et al., 2022)
PFCAs and PFSAs precursors, and PFECHS	Landfill Leachate	Queensland, Australia	Foam-fractionation (water treatment)	HPLC – MS/MS	PFCAs and PFSAs LOD = 0.02 – 0.05 µg/L, PFCAs and PFSAs LOQ = 0.08 – 0.17 µg/L, 6:2 FTS LOD = 0.03 µg/L, 6:2 FTS LOQ = 0.1 µg/L; PFECHS LOD = 0.03 µg/L, PFECHS LOQ 0.11 µg/L.	(Vo et al., 2023)
PFCAs and PFSAs precursors, TFA, FTIs, 6:2 Cl-PFESA, PFDeI, PFDoI PFBuDil, PFHxDil, PFODil. HFPO-DA	Atmosphere	Japan	Detection of PFASs in atmosphere	Combustion ion chromatography (CIC), HPIC, ILC-	IDL in pg, LOQ in pg, LOQ in pg/m^3: PFSAs = 0.003 – 3.67, 1.77 – 500, 0.033 – 9.43; PFCAs = 0.001 – 5.88, 1.89 – 500, 0.036 – 18.9; TFA = 0.836,200, 3.77; FTIs = 0.039 – 4.72, 50 – 1000,	(Lin et al., 2022)

				TQ MS with –ve ESI and GCMS	0.943 – 18.9; PFDeI = 1.20, 250, 4.72, PFDoI = 1.72, 250, 4.72 PFBuDil = 0.103, 50.0, 0.943, PFHxDil = 0.246, 50.0, 0.943, PFODil = 1.07, 250, 4.7; 6:2 Cl-PFESA = 0.005, 1.86, 0.035, HFPO-DA = 0.007, 2.00, 0.038	
PFCAs and PFSAs precursors, TFA, PFPA and many fluorinated transformation products due to degradation	Simulated water		Remediation of contaminated sites- photocatalysis	HPLC MS/MS For targeted analysis. UHPLC for non-targeted analysis	Detection limit was 5 ug/L for all samples, while quantification limits were set to lowest concentration of individual calibration curve	(Patch et al., 2022)
PFCAs and PFSAs precursors, GenX	Municial wastewater samples	Northern New Jersey, USA	Rapid analytical method for PFASs quantification	HPLC- TQMS and Nano-ESI-HRMS	LODs in ng/L first for Nano-ESI HRMS and then for HPLC-TQMS: PFSAs = 4.2 – 25.1, 1.1 – 135.6; PFCAs = 3.2 – 36.2, 6.7 – 87.8; GenX = 6.4, 96.6	(Wu et al., 2022)
PFCAs and PFSAs precursors, 6:2 Cl-PFESA, 8:2 Cl-PFESA, OBS, PF4OpeA, PF5OhxA, NaDONA, PFEESA, HFPO-DA, HFPO-TA	Surface water	Poyang Lake, China	Distribution, partitioning behavior and flux of PFAS	UPLC TQ in -ve ESI	MQL, MDL in pg/L for surface water: PFCAs = 0.41 – 18, 0.81 – 39; PFSAs = 1.4 – 33, 2.0 – 35; 6:2 Cl-PFESA = 1.8, 15, 8:2 Cl-PFESA = 1.0, 3.6; OBS = 2.0, 92; PF4OpeA = 6.8, 3.1; PF5OhxA = 3.0, 2.7; NaDONA = 3.0, 4.6; PFEESA = 0.94, n.d; HFPO-DA 6.9, 7.0, HFPO-TA = 11, 71	(Tang et al., 2022)

PFCAs, PFSAs precursors and many other using the non-targeted analysis	AFFF-impacted stormwater		Stormwater treatment-photocatalysis	LC-QtoF-MS. ICS-90 for fluoride. UHPLC for analyte.	MDL in ng/L for column: PFCAs = 400 – 950; PFSAs = 100 – 250.	(McIntyre et al., 2022)
PFCAs and PFSAs precursors, HFPO-DA, 6:2 Cl-PFAES, PFOAAmS, PFOSAmS, PFOAB, PFOSB and volatile decomposition products	Synthetic soils including clay and sand		Treatment of contaminated soil - pyrolysis and thermal air degradation	UPLC. Thermal desorption−pyrolysis (TD−Pyr) connected to a GC−MS to detect products of PFOA and PFOS	The limits of detection (S/N = 3) are 7 nmol/L for PFBA and PFPeA, 2 nmol/L for PFSAs, 5 nmol/L for other anionic PFAS, and 5 nmol/L for cationic and zwitterionic PFAS	(Alinezhad et al., 2022)
PFCAs and PFSAs precursors, Adona, GenX, PFOUDS.	Surface and tap waters	Biscayne Bay, USA	Presence of PFASs in water	LC-MS/MS system equipped with AJS ESI source	MDL, IQL in ng/L: PFCAs = 0.01 – 1.99, 0.26 – 205; PFSAs = 0.01 – 0.45, 0.51 – 97.3; Adona = 0.02, 0.98; GenX = 0.02, 5.90; PFOUDS = 0.36, 66.00.	(X. Li et al., 2022)
PFCAs and PFSAs precursors, ADONA, PFECHS, 6:2FTAB, 8-Cl-PFOS and PFOPA	Wastewater treatment plant influent	South-East Queensland, Australia	Trend of PFASs in WWTP influent	HPLC-MS/MS using TurboIonSpray® probe	PFCAs = 0.27 – 8.7; PFSAs = 0.54 – 32.0; ADONA = 1.0, PFECHS = 0.47, 6:2FTAB = 5.4, 8-Cl-PFOS = 1.0, PFOPA = 1.8.	(Gallen et al., 2022)
PFCAs and PFSAs precursors, 8:2Cl-PFESA, 6:2Cl-PFESA, PFECHS, HFPO-DA.	Drinking water source	Tianjin City, China	Detection of PFASs in lakes.	HPLC-MS/MS in negative ESI mode.	LOD, LOQ in ng/L: PFCAs = 0.02 – 0.14, 0.05 – 0.47; PFSAs = 0.00 – 0.03, 0.01 – 0.11; 8:2Cl-PFESA = 0.00, 0.01; 6:2Cl-PFESA = 0.00, 0.01; PFECHS = 0.00, 0.01; HFPO-DA = 0.08, 0.28.	(Y. Li et al., 2022)

HFPO-DA, PFCAs and PFSAs precursors	Spiked Synthetic tap water	University of Notre Dame, USA	New method for PFASs screening	PIGE Spectroscopy	LOD in ppt F, LOD in ppt analyte, HFPO-DA = 48.8, 77.1, PFSAs = 31.5 – 64.7, 34.6 – 59.4; PFCAs = 31.6 – 40, 45.9 – 58.8.	(Peaslee et al., 2021)
PFCAs, PFSAs, PFPiAs, diPAP precursors	Groundwater from contaminated site	Northern China plain	Determination of extent of ground water contamination	UHPLC-MS/MS TSQ Endura equipped with ESI	LODs, LOQs in ug/L: PFCAs = 0.003 – 0.05, 0.01 – 0.26; PFPAs = 0.028 – 0.138, 0.09 – 0.45; Precursors = 0.002 – 0.0935, 0.01 – 0.31; PFPiAs = 0.007 – 0.0165, 0.02 – 0.05; diPAP = 0.0056 – 0.0078, 0.02.	(J. Li et al., 2022)
PFCAs and PFSAs precursors, TFMS, PFMPA, PFMBA, PFEESA, NFDHA, HFPO-DA, and PFOPA	Biosolid extract and clean extract		New PFASs detection method	19F-NMR spectroscopy	A detection limit of 50 nM (25 ug/L for PFOS) was achieved in groundwater samples	(Camdzic et al., 2021)
PFCAs/PFSAs precursors, FhxSA, 6:2 FTS and other PFASs degradation by-products	AFFF impacted groundwater	Colorado, US	Water treatment- NF and UV-sulfite treatment train	LC-QtoF-MS	LOQ of Groundwater in ng/L: PFCAs = 0.7; PFSAs = 0.4 – 0.7; FhxSA = 0.4, 6:2 FTS = 0.7.	(Liu et al., 2021)
6:2 diPAP, 8:2 diPAP, Precursors of PFCAs/PFSAs and PFPA	House and fire station dust	US and Canada	Presence of PFASs in fire station and house dust	HPLC-ESI-MS/MS and GC/EI-MS	MDL in ng/g dust for house and fire station dust: 6:2 diPAP = 0.48, 2.54; 8:2 diPAP = 10.63, 9.63; PFCAs = 0.06 – 15.80, 0.47 – 48.90; PFSAs = 0.20 – 22.28, 0.97 – 8.56; PFPA = 0.14, 1.2.	(Hall et al., 2020)

PFCAs and PFSAs precursors, 6:2 Cl-PFESA, 8:2 Cl-PFESA, HFPO-DA, ADONA	Indoor dust from urban, industrial, and e-waste dismantling areas	Guangdong Province, China	Detection of PFASs in indoor dust of different indoor facilities	HPLC-MS/MS	LOQ of analytes ranged from 0.02 – 0.50 ng/mL	(B. Zhang et al., 2020)
Precursors of PFCAs, PFSAs and PFPAs, FHEA, FOEA, FOUEA, FDUEA, N-EtFHxSE (SYN 2), 6:6 PFPi, diSAmPAP HFPO-DA	Street sweepings	Across the US	Detection of PFASs in street sweepings	UHPLC-MS/MS	MDL, MQL in ng/g dust: PFCAs = 0.01 – 0.02, 0.01 – 0.69; PFSAs = 0.01 – 0.13, 0.03 – 0.42; PFPAs = 0.03 – 0.12, 0.10 – 0.41; HFPO-DA = 0.41, 1.37; FHEA = 0.08, 0.26; FOEA = 0.04, 0.13; FOUEA = 0.01, 0.05; FDUEA = 0.01, 0.03; N- 6:6 PFPi = 0.01, 0.04; diSAmPAP = 0.04, 0.12; EtFHxSE (SYN 2) = 0.40, 1.33.	(Ahmadireskety et al., 2021)
PFCAs and PFSAs (Linear and branched) precursors, NaDONA,	Dust from university buildings	US	To confirm implementation of "healthier material" manufactured without PFASs in buildings	HPLC-ESI-MS/ MS	MQL, MDL in ng/g: PFCAs = 0.05 – 9.19, 0.02 – 2.76; PFSAs = 0.05 – 15.06, 0.02 – 4.52; NaDONA = 0.10, 0.03.	(Young et al., 2022)
PFCAs and PFSAs precursors, 8:2 FTAcr, 10:2 FTAcr,	Air samples and carpets	Southern Rhodes Island, US	To study partitioning of volatile PFASs between air, dust and carpet	GC-MSD operating in positive chemical ionization mode using selected ion monitoring	LOQ in (ng/uL): PFCAs = 0.01 – 0.07; PFSAs = 0.002 – 0.20; 8:2 FTAcr = 0.01, 10:2 FTAcr = 0.02.	(Morales-Mcdevitt et al., 2021)

FTABs or FTSA-PrB, FTSASs, and FTSAS-So. Legacy PFASs including C6–C8 PFSAs, C4–C10 PFCAs. PFECHS, FtTAoS	Surface water	Melbourne, Australia	Detection from water as a result of fire event	LC-QtoF-MS	-	(Rana et al., 2022)
PFPrA, PFCAs, PFSAs, HFPO-DA	Soil samples near industrial area, airports, landfills and fire stations	Shanghai, China	Detection of PFASs contamination and distribution	LC-MS equipped with C18 column	MDL, MQL in ug/kg (dry weight): PFPrA = 0.05, 0.15; PFCAs = 0.02 – 0.1, 0.01 – 0.3; PFSAs = 0.003 – 0.03, 0.01 – 0.10; HFPO-DA = 0.003, 0.01.	(Zhu et al., 2022)
PFCAs, diPAP. PFSAs, DONA, GenX, 6:2 Cl-PFESA (F-53B)	Spiked tap water		Water treatment- Ozonation and GAC	HPLC-MS/MS	LOD, LOQ in ng/mL: PFCAs = 0.015 – 0.15, 0.03 – 0.30; diPAPs = 0.2 – 0.725, 0.40 – 1.45; PFSAs = 0.05 – 0.325, 0.10 – 0.65; DONA = 0.225, 0.45; GenX = 0.25, 0.50; CL-PFESA (F-53B) = 0.375, 0.75	(Kaiser et al., 2021)
Cl-PFPECA	Surface soil	New Jersey, USA	Predicting fate of new generation PFAS	HRUPLC-QtoF MS in –ve ESI for non-targeted analysis. LC-MS/MS for targeted analysis.	-	(Evich et al., 2022)
6:2 FTAB, 8:2 FTAB, 10: 2 FTAB, 5:1:2 FTB, 7:1:2 FTB, 9:1:2 FTB, 11:1:2 FTB, 5:3	Synthetic and surface water, Recycled wastewater	Vancouver Convention Centre, Canada	Drinking water treatment (adsorption)	LC-MS/MS<br>LC-HRMS (C18 columns) using ammonium acetate	-	(Dixit et al., 2021)

FTB, 5:3 FTB, 7:3 FTB, 9:3 FTB, 11:3 FTB.				or formic acid amended HPLC mobile phases		

[1] *measured as total PFAS.* [2] *Aqueous Film Forming Foam*. KEY: MDL = method detection limit, MQL = method quantification limit, LOD = limit of detection, LOQ = limit of quantification, IDL= instrument detection, IQL = instrument quantification limit, MRL = method reporting limit, I-LOD = instrument limit of detection, I-LOQ = instrument limit of quantification. PFCAs = Perfluoroalkyl carboxylic acids, PFSAs = Perfluoroalkyl sulfonic acids, FTAB = fluorotelomer sulfonamide betaine, FTB = fluorotelomer betaine, FTABs or FTSA-PrB = fluorotelomer sulfonamido betaines, FTSASs = fluorotelomer thioether amido sulfonic acids, FTSAS-So = fluorotelomer sulfonyl amido sulfonic acids, PFECHS = Perfluoro-4-ethylcyclohexanesulfonate, FtTAoS = fluorotelomer thioether amido sulfonic, PFBS = Perfluorobutanoic acid, PFHxS = Perfluorohexane sulfonate, PFOS = Perfluorohexane sulfonate, HFPO-DA = Hexafluoropropylene oxide-dimer acid, ADONA = dodecafluoro-3H-4,8-dioxanonanoate, 9C1-PF3ONS = 9-chlorohexadecafluoro-3-oxanone-1-sulfonic acid, NMeFOSAA1 = Methyl perfluorooctane sulfonamidoacetic acid, NEtFOSAA1= Ethyl perfluorooctane sulfonamidoacetic acid, 11C1-PF3OudS = 11-chloroeicosafluoro-3-oxaundecane-1-sulfonicacid, PFOA = Perfluorooctanoic acid , PFPrA = C3 perfluoropropanoic acid , PFPiAs = perfluorophosphinate , diPAP = Fluorotelomer phosphate diester, PF4OpeA = Perfluoro-4-oxapentanoic acid, PF5OhxA = Perfluoro-5-oxahexanoic acid, 3,6-OPFHpA = Perfluoro-3,6-dioxaheptanoic acid, TFA = trifluoroacetic acid, FTIs = diiodofluoroalkanes, PFDeI = perfluorodecyl iodide, PFDoI = perfluorododecyl iodide, PFBuDiI = 1,4-diiodoperfluorobutane, PFHxDiI = 1,6-diiodoperfluorohexane, PFODiI = 1,8-diiodoperfluorooctane, PFDA = Perfluorodecanoic acid, PFTeDA = Perfluorotetradecanoic acid, PFHpA = Perfluoroheptanoic acid, PFNA = Perfluorononanoic acid, PFPeS = Perfluoropentane sulfonate, PFPeA = Perfluoropentanoic acid, 6:2 Cl-PFESA = Chlorinated polyfluorinated ether sulfonate, 8:2 Cl-PFESA = Chlorinated polyfluorinated ether sulfonate , OBS = Sodium p -perfluorous nonenoxybenzenesulfonate, NaDONA = Sodium dodecafluoro-3H-4,8-dioxanonanoate, PFEESA = perfluoro (2-ethoxyethane)sulfonic acid, HFPO-TA = Hexafluoropropylene oxide trimer acid, C4-PFBA = Perfluorobutanoic acid, C6-PFHxA = Perfluorohexanoic acid, DONA = perfluoro-4,8-dioxa-3-nonanoic acid, Cl-PFPECA = chloro-perfluoropolyether carboxylates, Na-TFMS = sodium trifluoromethyl sulfonate, PFOPA = Perfluorooctyl phosphonic acid, 8:2-FTOH = Fluorotelomer alcohols, PTFE = Polytetrafluoroethylene, PFA = perfluoroalkoxy alkanes polymer, 4-FBA = 4-fluorobenzoic acid , TOLYLFL = tolylfluanid, 6:2 Cl-PFAES = 6:2 chlori-nated polyfluorinated ether sulfonate potassium salt, PFOAAmS = perfluorooctaneamido ammonium salt, PFOSAmS = perfluorooctanesulfonamidoammonium salt, PFOAB = perfluorooc-taneamido betaine, PFOSB =perfluor-ooctanesulfonamido betain, PFOUDS = Potassium 11-chloroeicosafluoro-3-oxaundecane-1-sulfonate, PFMPA = Perfluoro-3-methoxypropanoicacid, PFMBA = Perfluoro-4-methoxybutanoicacid, NFDHA = Nonafluoro-3,6-dioxaheptanoicacid, ,d-NMeFOSA = N-methyl-d3-perfluoro-1-octanesulfanamide, d-NEtFOSA, d9-NEtFOSA = N-ethylperfluoro-1-octanesulfonamide, d7-NMeFOSE = N-methyl-d7-perfluoro-1- sulfoamidoethanol. FhxSA, PFPA = perfluoroalkyl phosphinic acids, FHEA = 6:2 FTCA = 6:2 fluorotelomer carboxylic acids, FOEA = 8:2 FTCAs = 8:2 fluorotelomer carboxylic acids, FOUEA = 8:2 FTUCA = 8:2 unsaturated fluorotelomer carboxylic acids, FDUEA = 10:2 FTUCA = 10:2 unsaturated fluorotelomer carboxylic acids, N-EtFHxSE (SYN 2) = N-Ethyl-N-(2-hydroxyethyl) perfluorohexanesulfonamide, PFPi = perfluoroalkyl phosphinic acids, diSAmPAP = EtFOSE-based phosphate diester , FTAcr = Perfluorododecyl Acrylate, PFECA = polyfluoroalkyl ether carboxylic acids (GenX).
